# Hyperemesis gravidarum and the risk of emotional distress during and after pregnancy

**DOI:** 10.1007/s00737-017-0770-5

**Published:** 2017-08-25

**Authors:** Helena Kames Kjeldgaard, Malin Eberhard-Gran, Jūratė Šaltytė Benth, Åse Vigdis Vikanes

**Affiliations:** 1 HØKH, Akershus University Hospital, Post Box 1000, 1478 Lørenskog, Norway; 2 Institute of Clinical Medicine, Campus Ahus, University of Oslo, Lørenskog, Norway; 3 Division of Mental Health, Norwegian Institute of Public Health, Oslo, Norway; 4 The Intervention Centre, Oslo University Hospital, Oslo, Norway

**Keywords:** Depression, Emotional distress, Hyperemesis gravidarum, Mental health, Nausea and vomiting, Norwegian Mother and Child Cohort Study

## Abstract

Hyperemesis gravidarum (HG) is a pregnancy condition characterised by severe nausea and vomiting. Previous studies have shown an association between HG and depressive symptoms during pregnancy, but little is known about the risk of maternal psychological distress following an HG pregnancy. The objective of the current study was therefore to assess the association between HG and emotional distress during and after pregnancy. This was a population-based pregnancy cohort study using data from the Norwegian Mother and Child Cohort Study. A total of 851/92,947 (0.9%) had HG. Emotional distress was measured by the Hopkins Symptom Checklist (SCL-5) in gestational weeks 17 and 32 and 6 and 18 months postpartum. The generalised estimating equations model was estimated for assessing time trends in emotional distress. Adjustments were made for previous HG, lifetime history of depression, maternal age, parity, BMI, smoking before pregnancy, physical activity, length of education, and pelvic girdle pain. Women with HG had higher odds for emotional distress than women without HG at the 17th (*p* < 0.001) and 32nd gestational weeks (*p* = 0.001) in addition to 6 months postpartum (*p* = 0.005) but not 18 months postpartum (*p* = 0.430). Adjusted odds for emotional distress varied significantly over time for women with and without HG (*p* = 0.035). Women with HG were more likely to report emotional distress compared to women without HG during pregnancy and 6 months postpartum, but the difference between the groups disappeared 18 months after birth. The results suggest that the increased risk of developing emotional distress may primarily be a consequence of HG.

## Introduction

Nausea and vomiting in pregnancy (NVP) is common and affects up to 80% of all pregnancies, predominantly during the first trimester (Gadsby et al. 1993). Hyperemesis gravidarum (HG) is characterised by severe NVP starting before the 22nd week of gestation and can occur with or without metabolic disturbances (World Health Organization 2004). Due to the severity of NVP, HG is a main cause for sick leave (Dorheim et al. 2013) and hospitalisation during early pregnancy (Gazmararian et al. 2002), affecting between 0.3 and 2% of all pregnancies (Eliakim et al. 2000).

The aetiology of HG is unknown but has historically been explained by a variety of psychological disturbances or psychiatric diseases (Fairweather 1968). Today, HG is generally regarded as a disease of unknown pathophysiological origin (Grooten et al. 2015), although the opinion that HG is of psychological origin persists. Women with HG today report lack of support from their health care providers (Heitmann et al. 2016; Poursharif et al. 2008; Power et al. 2010), and those suffering from unremitting symptoms are still being evaluated for psychiatric conditions (Kim et al. 2009).

A recent meta-analysis showed an association between HG and depression and anxiety in pregnancy (Mitchell-Jones et al. 2016), but the direction of this association was not clarified. Some studies have found increased risk of HG in women with a history of depression (Fell et al. 2006; Kjeldgaard et al. 2017; Seng et al. 2007), whereas others found an increased risk of depression in HG women with no history of psychiatric disease (Aksoy et al. 2015; Pirimoglu et al. 2010). A relationship between the degree of nausea and vomiting and the risk of developing psychological distress including depression and anxiety has also been suggested (Koken et al. 2008; Kramer et al. 2013), but research shows conflicting results (Swallow et al. 2004; Tan et al. 2010). In addition, symptoms of anxiety and depression have been shown to decrease over time as symptoms of nausea and vomiting wear off (Annagur et al. 2013; McCarthy et al. 2011; Tan et al. 2014).

A number of studies have addressed psychological distress in the HG pregnancy (Mitchell-Jones et al. 2016), but little is known about the risk of maternal psychological distress following an HG pregnancy. Previous studies have in general been small or included limited information on possible confounders.

The aim of the present study was to assess whether HG was associated with emotional distress during pregnancy and up to 18 months after birth. The Norwegian Mother and Child Cohort Study (MoBa), comprising more than 100,000 pregnancies, provided a unique opportunity to explore this association.

## Materials and methods

### Study design and study population

From 1998 to 2008, all pregnant women scheduled to give birth at 50 of Norway’s 52 hospitals with maternity units received a postal invitation to participate in MoBa together with appointments for routine ultrasound examination at around week 17 of pregnancy. All participants signed an informed consent form (Magnus et al. 2016; Magnus et al. 2006). With the advantage of social stability and good health registries, Norway provides an excellent framework for a multisite, longitudinal cohort study. The current study was based on version 8 of the quality-assured data files linked to the Medical Birth Registry of Norway (MBRN), which is based on the compulsory notification of every birth or late abortion in Norway from week 16 of gestation, including information regarding pregnancy-related complications (Irgens 2000). Approximately 40% of the women who were invited participated, and each pregnancy was registered with a unique identification number.

The analyses of the current study are based on four questionnaires distributed in gestational weeks 17 (Q1) and 30 (Q2) and 6 (Q3) and 18 (Q4) months postpartum. Q1 addressed background factors including previous pregnancies, medical history before and during pregnancy, medication, occupation, exposures in the workplace and at home, lifestyle habits, and mental health. Q2 provided information about the mental and physical health at this stage of pregnancy as well as changes in habits and the work situation. The main themes in Q3 and Q4 were maternal physical and mental health as well as the child’s health, nutrition, and general development. English translations of the questionnaires can be found at http://www.fhi.no/moba.

The present study included all singleton pregnancies with known hospitalisation status. The study population consisted of 92,194 women, comprising 82% of the total sample.

### Variables

The main predictor was HG. HG was defined as prolonged nausea and vomiting leading to hospitalisation before the 25th gestational week, as reported in Q2 (week 30), in accordance with previous studies on MoBa data (Vikanes et al. 2010; Vikanes et al. 2013). Three quarters of HG cases were hospitalised during the first trimester in the MoBa. To clearly separate HG from less severe NVP, we restricted HG to comprise women who had been hospitalised due to this condition.

The main outcome was emotional distress, which was measured at all four time points; gestational weeks 17 and 30 and 6 and 18 months postpartum. A five-item short version (SCL-5) of the Hopkins Symptom Checklist-25 (SCL-25) was used as a proxy. The advantage of using the Hopkins Symptoms Checklist is that it was designed to measure symptoms of depression and anxiety in population surveys (Hesbacher et al. 1980, Strand et al. 2003). The SCL-5 is highly correlated with the SCL-25 (correlation coefficient of 0.92) (Tambs and Moum 1993) and consists of the following questions: Have you been bothered by any of the following during the last 2 weeks: (1) feeling fearful, (2) nervousness or shakiness inside, (3) feeling hopeless about the future, (4) feeling blue, and (5) worrying too much about things. The response categories ranged from “not bothered” to “very bothered” (range 1–4), with a maximum total score of 20. Symptoms of emotional distress were defined as a mean score > 2 (Strand et al. 2003), which has been shown to provide the same prevalence estimate of a depressive disorder as the Composite International Diagnostic Interview (Robins et al. 1988; Sandanger et al. 1998). In the current sample, the SCL-5 had adequate internal consistency with a Cronbach’s alpha of 0.78 at gestational week 17, 0.81 at gestational week 30, 0.82 at 6 months postpartum, and 0.82 at 18 months postpartum. The SCL-5 has been used in several previous studies as a measurement for emotional distress (Eriksen et al. 2006, Tambs et al. 2009).

Missing values in the dichotomised version of the SCL-5 were handled as follows. First, the average score of existing items was calculated for each case if at least three of the five questions were answered. If the average of the existing items was clearly above or below the cut-off and could not be affected by imputation of missing values, it was dichotomised to zero or one, as appropriate. Imputation was not performed in cases where the average score did not uniquely define the value above or below cut-off. Altogether *N* = 25 cases were imputed.

Covariates obtained from the MBRN included maternal age and parity, while possible confounders obtained from MoBa Q1 were previous HG (Trogstad et al. 2005), previous depression, socioeconomic status, BMI, smoking (Fell et al. 2006; Lancaster et al. 2010; Vikanes et al. 2010), and physical activity pre-pregnancy (Haakstad et al. 2016). The possible confounder from MoBa Q2 was pelvic girdle pain (Bjelland et al. 2013; Chortatos et al. 2015).

Previous HG was assessed as a yes/no response to the question “Have you had any of the following problems during previous pregnancies: serious nausea and vomiting?”

Previous depression was measured using the Kendler’s life-time major depression scale (KLTDS) (Kendler et al. 1993). The KLTDS was defined using five of the nine symptomatic criteria for major depression in DSM-III-R: Have you ever experienced the following for a continuous period of 2 weeks or more: (1) felt depressed, sad; (2) had problems with appetite or eaten too much; (3) been bothered by feeling weaker or a lack of energy; (4) really blamed yourself and felt worthless; and (5) had problems with concentration or had problems making decisions. The response to each question was yes or no, and a history of depression was defined as present if a minimum of three of the five symptoms and sad mood were reported to occur simultaneously for more than 2 weeks.

Parity was dichotomised as either primiparous or multiparous. Pre-pregnancy body mass index (BMI) was calculated as weight/height^2^. Women were excluded if they were shorter than 120 cm (*n* = 199) and weighed more than 150 kg or less than 40 kg (*n* = 58). Women reporting a weight reduction of more than 20 kg or an increase of more than 50 kg since the start of pregnancy were also excluded (*n* = 65). Smoking was assessed as a yes/no response to the question “Did you smoke three months before pregnancy?” Education was used as a proxy for socioeconomic status, and the length of education (in years) was divided into two categories. Pelvic girdle pain was defined as pain in the anterior pelvis and on both sides in the posterior pelvis. Pre-pregnancy physical activity was divided into three categories: never, one to three times a month, and one to two times per week or more.

Thyroid disease was not included in the analysis as the questionnaire form does not allow differentiation between hyperthyroid and hypothyroid disease.

### Statistical analysis

Data were described as means and standard deviations (SD) or frequencies and percentages, as appropriate. To assess the association between HG and symptoms of emotional distress measured twice during the pregnancy and twice postpartum, the generalised estimating equations (GEE) model adjusting for intra-women correlations due to repeated measurements was estimated. The model contained random effects for women and fixed effects for time component up to second order, HG, and the interaction between the two. A significant interaction would imply that the development of emotional distress during pregnancy and postpartum is of different character among those with and without HG. The time trend was further adjusted for a number of potential confounders. To avoid hypothesis fishing, a data splitting approach was applied (Dahl et al. 2008). According to this approach, the data set was split into two random parts containing approximately 30 (part I) and 70% (part II) of observations. Splitting was performed within stratas defined by several key variables. Part I (pilot) was used to construct a model for HG. Only predictors significant at the 5% level or those otherwise considered important were left in the model estimated on pilot data. The hypothesis testing was then performed on part II (test) data. Only the results with *p* values below 0.05 in the test data analyses were accepted as significant, regardless of significance level in the pilot part. Once the hypotheses were tested, the model was estimated on the entire data set to achieve most accurate estimates for the model parameters. Due to the numerous predictors considered, the level of significance was set to 0.005 when interpreting the results in the entire data set.

The results were tabulated as regression coefficients, standard errors (SE), and corresponding *p* values. For easier interpretation, unadjusted and adjusted odds ratios (OR) with the corresponding 95% confidence intervals (CI) were derived within each group at every time point and presented graphically.

All analyses were performed in IBM SPSS Statistics for Windows, Version 22.0, Armonk, NY.

## Results

Among the 92,947 women included in this study, the mean age was 30.2 years (14–47 years; SD 4.5 years) and 45.5% were primiparous. A total of 851 (0.9%) women reported hospitalisation due to nausea and vomiting during the index pregnancy. Frequencies of emotional distress at the four time points are shown in Table 1.

**Table 1:** Score of emotional distress at different time points according to presence of hyperemesis gravidarum (HG) among 92,947 pregnant women

	Low distress score *n* (%)	High distress score *n* (%)	Total *n*
Gestational week 17			
No HG	83,371 (99.1)	5965 (98.3)	89,336
HG	729 (0.9)	101 (1.7)	830
Total	84,100	6066	90,166
Gestational week 32			
No HG	85,258 (99.1)	6091 (98.4)	91,349
HG	746 (0.9)	99 (1.6)	845
Total	86,004	6190	92,194
6 months postpartum			
No HG	77,336 (99.1)	4990 (98.4)	82,326
HG	677 (0.9)	81 (1.6)	758
Total	78,013	5071	83,084
18 months postpartum			
No HG	65,013 (99.1)	5131 (98.7)	70,144
HG	560 (0.9)	68 (1.3)	628
Total	65,573	5199	70,772

Covariates included in the regression model are presented in Table 2. Previous HG, history of depression, shorter education, smoking, pelvic girdle pain, low pre-pregnancy physical activity, and high pre-pregnancy BMI were positively associated with symptoms of emotional distress. Maternal age was negatively associated with symptoms of emotional distress.

**Table 2:** Characteristics of the sample according to emotional distress status at gestational week 17 among 90,166 women

	Low distress score *n* (%)	High distress score *n* (%)	Total *n*
Previous HG			
No	68,656 (93.7)	4637 (6.3)	73,293
Yes	15,428 (91.5)	1428 (8.5)	16,856
History of depression			
No	65,318 (96.7)	2219 (3.3)	67,537
Yes	15,346 (83.0)	3145 (17.0)	18,491
Parity			
Primipara	38,018 (92.7)	2979 (7.3)	40,997
Multipara	46,082 (93.7)	3087 (6.3)	49,169
Length of education (years)			
≤ 16	5266 (84.7)	954 (15.3)	6220
> 16	74,784 (94.1)	4688 (5.9)	79,472
Physical activity			
Never	607 (86.3)	96 (13.7)	703
One to three times per month	6470 (91.9)	569 (8.1)	7039
One to two or more times per week	75,295 (93.5)	5222 (6.5)	80,517
Smoking			
No	51,424 (94.7)	2853 (5.3)	54,277
Yes	22,018 (89.2)	2653 (10.8)	24,671
Pelvic girdle pain^a^			
No	72,096 (93.9)	4658 (6.1)	76,754
Yes	12,004 (89.5)	1408 (10.5)	13,412
	Low distress score	High distress score	Total
Maternal age			
*N*	84,100	6066	90,166
Mean (SD)	30.3 (4.5)	28.9 (5.3)	30.2 (4.5)
Pre-pregnancy BMI			
*N*	81,972	5873	87,845
Mean (SD)	24.0 (4.22)	24.3 (4.80)	24.0 (4.3)

^a^Information about pelvic girdle pain was obtained in gestational week 32

**Fig. 1:**
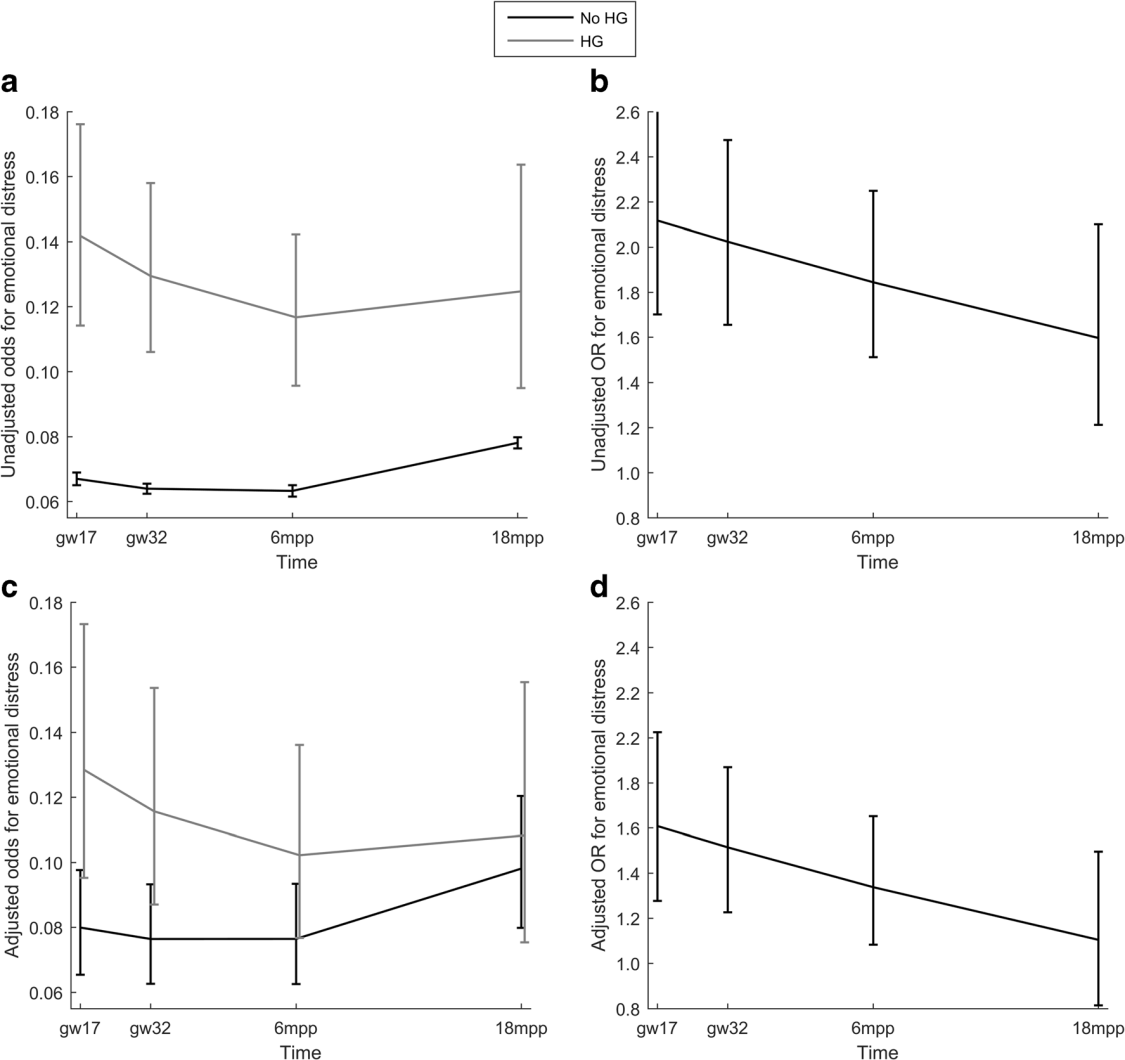
Time profiles of unadjusted and adjusted odds (**a** and **c**) and odds ratios (**b** and **d**) for emotional distress according to presence of HG estimated by a GEE model at gestational weeks (gw) 17 and 32 and 6 and 18 months postpartum (ppm)

Time trend analyses (Table 3) revealed a non-linear relationship between HG and emotional distress. The time profiles in adjusted odds for emotional distress were significantly different between HG and no HG groups (*p* = 0.035). The odds for emotional distress (Fig. 1) were statistically significant between HG and no HG groups at the 17th (*p* < 0.001) and 32nd gestational week (*p* = 0.001) in addition to 6 months postpartum (*p* = 0.005) but not 18 months postpartum (*p* = 0.430).

**Table 3:** Unadjusted and adjusted GEE model for time profile of emotional distress among 69,200 women

Variable	Unadjusted model		Adjusted model	
	Regr. coeff. (SE)^a^	*p* value	Regr. coeff. (SE)^a^	*p* value
Time (weeks)	−0.004 (0.0007)	< 0.001	−0.004 (0.0008)	< 0.001
Time^2^	0.00006 (0.000007)	< 0.001	0.00007 (0.000008)	< 0.001
HG				
No	0		0	
Yes	0.750 (0.111)	< 0.001	0.475 (0.118)	< 0.001
Time × HG				
HG no	0		0	
HG yes	−0.003 (0.002)	0.029	− 0.004 (0.002)	0.035
	OR (95% CI)	*p* value	OR (95% CI)	*p* value
Previous HG				
No	1		1	
Yes	1.40 (1.33; 1.47)	< 0.001	1.26 (1.19; 1.33)	< 0.001
History of depression				
No	1		1	
Yes	5.06 (4.84; 5.28)	< 0.001	4.81 (4.60; 5.02)	< 0.001
Maternal age	0.952 (0.947; 0.958)	< 0.001	0.956 (0.951; 0.961)	< 0.001
Parity				
Primipara	1		1	
Multipara	0.93 (0.89; 0.97)	0.001	0.97 (0.92; 1.02)	0.214
Length of education (years)				
≤ 16	2.53 (2.37; 2.71)	< 0.001	1.75 (1.63; 1.88)	< 0.001
> 16	1		1	
Physical activity				
Never	2.12 (1.75; 2.57)	< 0.001	1.56 (1.28; 1.91)	< 0.001
One to three times per month	1.22 (1.13; 1.32)	< 0.001	1.06 (0.98; 1.15)	0.127
One to two or more times per week	1 1.02 (1.016; 1.027)	– < 0.001	1 1.012 (1.007; 1.017)	< 0.001
Pre-pregnancy BMI Smoking				
No	1		1	
Yes	1.91 (1.83; 2.00)	< 0.001	1.48 (1.42; 1.56)	< 0.001
Pelvic girdle pain				
No	1		1	
Yes	1.88 (1.78; 1.98)	< 0.001	1.47 (1.39; 1.55)	< 0.001

^a^Regression coefficient and standard error with odds ratios (OR) for emotional distress presented in Fig. 1 for those with and without HG at each time point

In subanalyses, we assessed whether having been hospitalised for HG in the first trimester only, the second trimester only, or in both trimesters had different risk profiles for developing emotional distress. The analyses were adjusted for the same covariates as the main analyses. The results showed no difference in odds for emotional distress between women who had been hospitalised in the first trimester and women who had been hospitalised in the second trimester. There was also no difference between women who had been hospitalised in one trimester only compared to those without HG. However, women who had been hospitalised in both trimesters had significantly higher odds for symptoms of emotional distress compared to women hospitalised in the first or second trimester only (data not shown).

## Discussion

In the present study, including 92,947 women, we assessed the development of emotional distress over time from the 17th gestational week up to 18 months after birth, depending on whether the women suffered from hyperemesis or not. At the 17th and 32nd gestational weeks and at 6 months postpartum, women with HG had higher odds for emotional distress compared to women without HG. In contrast, at 18 months postpartum, there was no difference in odds for emotional distress between the two groups.

Several previous studies have reported decreasing psychological distress over the course of pregnancy in women with HG. Annagur et al. (2013) evaluated 47 women with HG for psychiatric disease in all three trimesters. They found that the number of women with anxiety and depression was reduced as the nausea and vomiting resolved and the pregnancy progressed. Unfortunately, a comparison group was not included, which explains why it is not possible to claim that women with HG were more likely to develop anxiety and depression than women without. Tan et al. (2014) found that the psychological distress in 121 women with HG decreased from the first to the third trimester along with declining symptoms of nausea and vomiting. In the third trimester, the degree of psychological distress was even lower in the HG group than that in the comparison group. The authors therefore argued that the psychological distress observed in women with HG is self-limiting and a probable result of HG. There was, however, no data available on the psychological distress in the comparison group during the first trimester. In a study on 164 hyperemetic women, McCarthy et al. (2011) also reported decreasing psychological distress during pregnancy in women with HG. Although the psychological distress normalised when symptoms of HG improved, the psychological burden was reported to be increased for weeks after cessation of HG. Additionally, they found that women with HG had higher odds of depression at gestational weeks 15 and 20 compared to women without HG.

Senturk et al. (2017) reported that among the 207 HG women studied, there was an increased risk of depression during not only the first trimester but also postpartum (unadjusted OR 6.52 (95% CI 3.77–11.30)). The time point for assessing postpartum depression was, however, not specified. Additionally, the women were not evaluated during the second or third trimester.

Previous research has shown contradictory results regarding whether the timing and duration of HG may affect the risk of psychological distress. McCarthy et al. (2011) revealed that women with ongoing vomiting after the 15th gestational week were more likely to have depression at 20 weeks compared to HG women whose vomiting had ceased before the 15th week. On the other hand, Tan et al. (2010) found no association between psychological distress and duration of vomiting prior to hospitalisation. In the present study, we therefore assessed whether having been hospitalised for HG in the first trimester only, the second trimester only, or in both trimesters had different risk profiles for emotional distress. Results showed that women who had been hospitalised due to HG in both trimesters had significantly higher odds for subsequent emotional distress compared to women who had been hospitalised in one trimester only. The results suggest that prolonged duration of HG symptoms increases the risk of emotional distress.

To our knowledge, no previous study has estimated the associations between HG and emotional distress at several time points during pregnancy and up to 18 months after delivery. Our results suggest that psychological symptoms during the HG pregnancy and postpartum period may reflect the severity as well as the duration of HG symptoms and therefore might resolve gradually following the cessation of HG. In a previous study (Kjeldgaard et al. 2017), we found that a life-time history of depression was associated with a 50% increase in risk of HG, but that most women with HG did not have a history of depression. Hence, we did not consider a history of depression to be a main driver in the pathogenesis of HG. In the present study, we hypothesised that if women with HG are more likely to be distressed about their lives in general, we would expect a higher risk of emotional distress at all four time points. Although the current study cannot be used to determine causation, the fact that the difference in emotional distress between women with HG and women without HG decreased over time suggests that the greater risk of emotional distress seen in women with HG may be due to the severity as well as duration of their symptoms. This suggestion is further supported by our finding that it was the women who were hospitalised in both trimesters, in particular, who had a significantly greater risk of emotional distress. Our results advocate that health care providers focus first and foremost on treating dehydration and nutritional deficiencies before possible emotional distress in order to ensure the health of the mother and foetus, as inadequate care may have severe consequences including therapeutic abortions, Wernicke’s encephalopathy, and even death (Eliakim et al. 2000; Poursharif et al. 2007).

An advantage of the MoBa study is the availability of information on a history of depression. Several previous studies did not have such information and could therefore not address this question (McCarthy et al. 2011) or did not include women with previous depression (Mitchell-Jones et al. 2016). In the current study, a history of depression was the strongest risk factor (OR 3.42, 95% CI (3.29; 3.56)) for developing emotional distress during and after pregnancy. This was not surprising as a large body of research has shown that a history of depression is a main risk factor for prenatal and postpartum depressive symptoms (Biaggi et al. 2016, Norhayati et al. 2015). Having had a previous HG pregnancy is known to increase the risk of developing HG in a following pregnancy (Trogstad et al. 2005). We therefore adjusted for previous HG in the time trend analyses as women who had HG previously may be more susceptible to develop emotional distress in a current HG pregnancy. We found an adjusted OR of 1.18, 95% CI (1.12; 1.24). In agreement with previous studies, we found a positive association between emotional distress and younger age of the mother, higher pre-pregnancy BMI, shorter education, low physical activity, and pelvic girdle pain, whereas parity had a protective effect on the risk of developing emotional distress.

In the present study, we used an instrument (SCL-5) designed to measure psychological distress in population-based studies (Strand et al. 2003). It has been validated in several populations and is documented as an acceptable screening instrument for depression as defined by ICD-10 (Sandanger et al. 1998). In the MoBa study, the women’s answers to SCL-5 were the only available measures of mental health status. Generally, questionnaire studies enable larger study populations than studies utilising clinical interviews for data collection. A clinical interview, however, remains the gold standard for assessing depression and anxiety. Furthermore, it is important to note that SCL-5 is a screening tool for depressive and anxious symptoms and cannot be used for diagnosing depression or anxiety. Extensive questionnaire studies with a broad scope such as the MoBa study often have space limitations for the original lengthy psychometric instruments, and short versions may be useful to improve response rates. While the short versions affect measurement precision, the level of precision remains sufficient for epidemiological purposes (Strand et al. 2003; Tambs and Moum 1993; Tambs and Røysamb 2014).

The large number of HG pregnancies (*n* = 851) was a major strength of the current study. In addition, the study covered all regions of Norway, and the risk of recall bias was reduced by the prospective nature of data collection. To date, more than 400 articles have been published based on MoBa data (Magnus et al. 2016). However, some limitations may be considered. Self-selection bias might be present as only about 40% of the invited women participated in the study. However, the potential bias by skewed selection of participants in MoBa may influence the prevalence estimates rather than the exposure-outcome associations (Nilsen et al. 2009). Women known to be underrepresented in MoBa included immigrants, smokers, single women, those with shorter education, and those less than 25 years of age (Nilsen et al. 2009; Vikanes et al. 2010).

Another limitation concerns the definition of HG. Unfortunately, the MoBa does not include a validated score such as the Pregnancy Unique Quantification of Emesis (PUQE) (Birkeland et al. 2015; Koren et al. 2005) to assess the symptoms of nausea and vomiting. The data provide information about the weeks of pregnancy in which women suffered from nausea and vomiting and whether or not these women were hospitalised due to NVP. In Norway, only women with severe metabolic disturbances are admitted to the hospital for HG, and no outpatient treatment is available. Health care is free of charge in Norway, and all women in need of inpatient treatment are admitted regardless of socioeconomic status. Therefore, in order to restrict our HG group to those with the most severe symptoms, we defined HG as hospitalisation due to nausea and vomiting. As such, our conclusions can be applied only to women with severe symptoms of HG. Although hospitalisation for HG was assessed retrospectively, recall bias was highly unlikely due to the relatively short interval between hospitalisation and reporting of HG in week 32 of pregnancy (Vikanes et al. 2010).

Unfortunately, we did not have data on emotional distress during the first trimester, which is a limitation of our study. To our knowledge, no previous population-based study has succeeded in studying mental distress when HG patients are at the peak of their symptoms. The reason for this may be that the most severely affected women at that point in time are in critical condition clinically, as characterised by exhaustion due to dehydration and nutritional deficiencies.

Furthermore, regarding concurrent diseases or conditions, the comparison group comprised all other pregnant women in the study, including those with complications other than HG, thus reducing the risk of overestimating the association between HG and depression. Thyroid diseases are associated with HG as well as a variety of emotional disturbances and may hence be an important confounder to adjust for. Unfortunately, the data did not distinguish between hyperthyroid and hypothyroid diseases, and therefore we did not adjust for these conditions. This may have affected our results.

Our results should be interpreted with these limitations in mind.

## Conclusion

Women with an HG pregnancy were more likely to report emotional distress than women without HG, but the differences between these groups disappeared 18 months after birth. Subanalyses showed that women with prolonged HG were more likely to develop symptoms of emotional distress. The findings suggest that higher risks of developing emotional distress may primarily be a consequence of HG.
